# Health system context and implementation of evidence-based practices—development and validation of the Context Assessment for Community Health (COACH) tool for low- and middle-income settings

**DOI:** 10.1186/s13012-015-0305-2

**Published:** 2015-08-15

**Authors:** Anna Bergström, Sarah Skeen, Duong M. Duc, Elmer Zelaya Blandon, Carole Estabrooks, Petter Gustavsson, Dinh Thi Phuong Hoa, Carina Källestål, Mats Målqvist, Nguyen Thu Nga, Lars-Åke Persson, Jesmin Pervin, Stefan Peterson, Anisur Rahman, Katarina Selling, Janet E. Squires, Mark Tomlinson, Peter Waiswa, Lars Wallin

**Affiliations:** 1 International Maternal and Child Health, Department of Women’s and Children’s Health, Uppsala University, Uppsala, Sweden; 2 Department of Psychology, Stellenbosch University, Stellenbosch, South Africa; 3 Hanoi School of Public Health, Hanoi, Vietnam; 4 Fundacion Coordinación de Hermanamientos e Iniciativas de Cooperación CHICA, León, Nicaragua; 5 Faculty of Nursing, University of Alberta, Edmonton, Canada; 6 Department of Clinical Neuroscience, Division of Psychology, Karolinska Institutet, Stockholm, Sweden; 7 Research Institute for Child Health, National Hospital of Paediatrics, Hanoi, Vietnam; 8 Centre for Reproductive Health, International Centre for Diarrhoeal Disease Research, Dhaka, Bangladesh; 9 Department of Public Health Sciences, Karolinska Institutet, Stockholm, Sweden; 10 School of Public Health, Makerere University College of Health Sciences, Kampala, Uganda; 11 Ottawa Hospital Research Institute, Ottawa, Canada; 12 School of Nursing, Faculty of Health Sciences, University of Ottawa, Ottawa, Canada; 13 School of Education, Health and Social Studies, Dalarna University, Falun, Sweden; 14 Department of Neurobiology, Care Sciences and Society, Division of Nursing, Karolinska Institutet, Stockholm, Sweden

## Abstract

**Background:**

The gap between what is known and what is practiced results in health service users not benefitting from advances in healthcare, and in unnecessary costs. A supportive context is considered a key element for successful implementation of evidence-based practices (EBP). There were no tools available for the systematic mapping of aspects of organizational context influencing the implementation of EBPs in low- and middle-income countries (LMICs). Thus, this project aimed to develop and psychometrically validate a tool for this purpose.

**Methods:**

The development of the Context Assessment for Community Health (COACH) tool was premised on the context dimension in the Promoting Action on Research Implementation in Health Services framework, and is a derivative product of the Alberta Context Tool. Its development was undertaken in Bangladesh, Vietnam, Uganda, South Africa and Nicaragua in six phases: (1) defining dimensions and draft tool development, (2) content validity amongst in-country expert panels, (3) content validity amongst international experts, (4) response process validity, (5) translation and (6) evaluation of psychometric properties amongst 690 health workers in the five countries.

**Results:**

The tool was validated for use amongst physicians, nurse/midwives and community health workers. The six phases of development resulted in a good fit between the theoretical dimensions of the COACH tool and its psychometric properties. The tool has 49 items measuring eight aspects of context: Resources, Community engagement, Commitment to work, Informal payment, Leadership, Work culture, Monitoring services for action and Sources of knowledge.

**Conclusions:**

Aspects of organizational context that were identified as influencing the implementation of EBPs in high-income settings were also found to be relevant in LMICs. However, there were additional aspects of context of relevance in LMICs specifically Resources, Community engagement, Commitment to work and Informal payment. Use of the COACH tool will allow for systematic description of the local healthcare context prior implementing healthcare interventions to allow for tailoring implementation strategies or as part of the evaluation of implementing healthcare interventions and thus allow for deeper insights into the process of implementing EBPs in LMICs.

**Electronic supplementary material:**

The online version of this article (doi:10.1186/s13012-015-0305-2) contains supplementary material, which is available to authorized users.

## Background

The 2012 World Health Report *No Health Without Research* emphasized the importance of implementing research into policy and practice as a means of achieving universal and equitable access to healthcare [1]. This highlights the challenges to determine the most effective implementation strategies for interventions, how to understand which strategies work where and why, and in doing so, promoting the better use of research [2–4].

In order to move from ‘what works’ to ‘what works where and why’, there is a need to generate evidence of what facilitates successful implementation. The Promoting Action on Research Implementation in Health Services (PARIHS) framework was developed to provide a framework to understand implementation as a multifaceted process [5]. The framework emphasizes the strength of and interplay between the following: (a) the nature of the *evidenc*e being used, (b) the quality of the *context* in terms of coping with change and (c) the *facilitation* relevant for a successful change process [6, 7]. Thus, in addition to the availability of evidence for a certain innovation or practice and facilitation as a strategy used to implement this evidence, the context in which the evidence is implemented matters. Hence, there is a need to go beyond measuring the ‘hardware’ of the health system to capturing the ‘software’, i.e. contextual issues, including the ideas, values, norms and power relations that determine health system performance [8]. Context, in relation to implementing EBPs in healthcare settings, has been defined as ‘the environment or setting in which the proposed change is to be implemented’ [5]. Understanding the socio-political nature of health systems, the organization’s readiness to change and the role of tailored implementation is regarded as a priority field in implementation science, including the need to systematically study the attributes of context influencing this process [9–16].

The importance of understanding context prior to and during the evaluation of the implementation of EBPs has led to the development of three quantitative tools aimed at assessing healthcare context, all of which have been developed based on the PARIHS framework [17–19]. Out of the three tools, the Alberta Context Tool (ACT) is the one that has been most widely used and has been subjected to the most rigorous evaluation of validity and reliability [13, 20–24]. The tool was developed in Canada, has been psychometrically tested also in other countries and is presently used in several large studies in high-income settings [20, 25–27]. The ACT contains eight dimensions measuring (1) leadership, (2) culture, (3) feedback, (4) connection amongst people, (5) formal interactions, (6) informal interactions, (7) structural and electronic resources and (8) organizational slack (sub-divided into staffing, space and time) [19].

The three available tools were developed for, and validated in, high-income settings [17–19, 25, 26]. There has been no tool readily available for use in low- and middle-income countries (LMICs), where contextual issues influencing efforts to implement EBPs might include other aspects than those in high-income settings [28, 29]. The objective of the Context Assessment for Community Health (COACH) project was to develop and psychometrically validate a tool for LMICs to assess aspects of context influencing the implementation of evidence-based practices (EBP) [30] that could be used to achieve better insights into the process of implementing EBPs. The name of the tool was chosen to reflect the focus of the project in terms of understanding how health systems context relates to the provision of care to community members. The purpose of the tool is to (1) enhance the opportunities to act on locally identified shortcomings of the health system to increase effectiveness, (2) guide planning and promote adaptation of implementation strategies to the local context and (3) link contextual characteristics to outcome indicators of healthcare interventions. Out of the three developed tools developed for high-income settings, the Organizational Readiness to Change Assessment [17] assesses all three components of the PARIHS model, i.e. evidence, facilitation and context, and the Context Assessment Index [18] has a stronger focus on the individual health worker. Thus, the ACT, which has a stronger focus on assessing organizational aspects of context were perceived to be a suitable tool to depart from. Also, similarly to the ACT, we aimed to develop a tool that focused on modifiable aspects of context, i.e. that could be intervened upon [19].

## Methods

This project was developed within an informal network, which had a focus on implementation research and the ‘know-do-gap’ in relation to the millennium development goals 4–6 [31]. In a network meeting in 2010, a member of the network identified the need for a tool to assess local organizational context in LMICs as context was seen as an important variable influencing the implementation of health interventions. Thus, the network formed a core group to carry out the COACH project including health services researchers from Bangladesh, Vietnam, Uganda, South Africa, Nicaragua and Sweden having extensive experience from working in LMICs and implementing EBPs in these settings. Early in the development, we initiated collaboration with two Canadian researchers who lead the development of the ACT. The rationale for forming this multi-country team was our common interest in context as an explanatory factor influencing the implementation of health interventions.

### Study settings and design

**Fig. 1:**
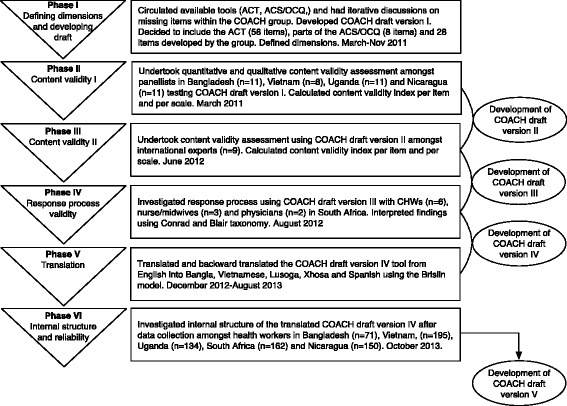
Summary of the COACH tool development

The COACH tool development has gone through six different phases resulting in five different versions of the COACH tool (see Fig. 1). Findings from one phase fed into the development of the next version of the tool including deletion of items, revisions of items and development of new items. Our approach was guided by the *Standards for Educational and Psychological Testing* [32] considered best practice in the field of psychometrics. Study sites were involved at similar stages of the project: Bangladesh (phases II, V, VI); Vietnam (phases II, V, VI); Uganda (phases II, V, VI); South Africa (phases IV, V, VI); and Nicaragua (phases II, V, VI).

### Ethical approvals

Ethical approval was obtained from the Ethical Review Committee of the International Centre for Diarrhoeal Disease Research, Bangladesh (icddr,b), the Ethical Scientific Committee at Ministry of Health in Vietnam, León Medical Faculty Ethical Board in Nicaragua, Health Research Ethics Committee at Stellenbosch University in South Africa and Uppsala Regional Ethical Review Board in Sweden. In Uganda, ethical approval was obtained from the Makerere University School of Public Health Institutional Review Board and Uganda National Council of Science and Technology.

### Phase I: defining dimensions and developing a draft version of the COACH tool

Defining the main dimensions (constructs) to be measured by the tool and developing items under each dimension was a key first step in the development of the COACH tool [33]. The process was iterative in that it was initiated during phase I, but further informed and revised by the findings from latter phases.

### Dimensions

Initially, we reviewed studies focusing on how context influences the implementation of EBPs in healthcare [28, 29, 34–42], and the interconnected health system building blocks as presented by the WHO [43]. Furthermore, we considered and concluded that the ACT was a suitable starting point. Based on an agreement with the developers of the ACT, we thereinafter initiated the process of identifying constructs to be included in the new tool by reviewing dimensions in the ACT, which were found to have good psychometric properties in different settings [20–22]. Following that, we identified aspects of context that were not explicitly stated in the ACT dimensions, but perceived to be of relevance for a tool for LMICs.

### Items

We agreed that all dimensions in the ACT were relevant for testing in LMIC settings, and thus, all ACT items were included in the first version of the COACH tool. In order to capture re-defined and newly developed dimensions, we went through a process of developing items based on these new dimensions through iterative discussions.

Phase I resulted in the development of COACH version I tool.

### Phase II: testing content validity in country panels

Content validity allows for an identified panel of 8–12 individuals to assess the perceived relevance of each item in a tool [44]. Experts rated the relevance of each item (*n* = 94 items) in the COACH tool as 1 = not relevant, 2 = somewhat relevant, 3 = quite relevant and 4 = highly relevant [44]. Based on panels’ assessments, the item-content validity index (I-CVI), a measure of the proportion of raters in agreement of relevance per item and two types of scale-content validity indices (S-CVI), measuring the relevance of each dimension [44, 45] were calculated.

In phase II, content validity assessment was undertaken using the English version of the COACH version I tool amongst identified panel subjects in Bangladesh (*n* = 11), Vietnam (*n* = 8), Uganda (*n* = 11) and Nicaragua (*n* = 11). Panels included participants with contextual expertise and with the following characteristics: (1) researchers/managers or programmers with experience of large-scale implementation of healthcare interventions and (2) health workers in decision-making positions with experience of leading implementation of healthcare interventions. To explore additional factors that might influence implementation of EBPs in the current settings, we conducted focus group discussions with each of the panels on the content of the tool (see Table 1 for the semi-structured guide).

**Table 1:** The focus group discussion guide used following the individual content validity testing in Bangladesh, Vietnam, Uganda and Nicaragua, phase II

*1. In terms of uptake of new knowledge, what are your thoughts around this dimension as a whole?*
*2. How well do you find these items to reflect the dimension as a whole?*
*3. If we look at the individual items, what are your thoughts about them?*
*4. Do you believe that any of the three targeted types of healthcare providers will find it difficult to answer to any of the items in the dimension and if so, why?*
*5. Are there any aspects you missed? As you read these items now, do you see any reason to remove any item? Would it require any other item? What would it then cover?*

A COACH version II tool was developed based on findings from phase II.

### Phase III: testing content validity in an international panel

Using a similar method to the content validity procedure in Phase II, we undertook a second set of content validity assessments with this version with eight international experts with extensive experience in undertaking research on the implementation of health interventions in LMICs. Content validity was assessed using an online console where experts could enter additional comments about each dimension in addition to rating the perceived relevance of items.

A COACH version III tool was developed based on findings from phase III.

### Phase IV: investigating and analysing the response process validity

To understand how target group respondents perceived and understood the COACH version III tool, we investigated the response process with community health workers (CHWs) (*n* = 6), nurse/midwives (*n* = 3) and physicians (*n* = 2) in South Africa. Response process interviews using the ‘think aloud’ method [46] were conducted in English in order to achieve a solid English version of the tool ahead of translations. The method implies that respondents verbally report their thinking whilst answering survey questions [47]. Response process data were analysed using Conrad and Blair’s taxonomy [47] addressing the following: lexical problems, inclusion/exclusion problems, temporal problems, logical problems and computational problems.

A COACH version IV tool was developed based on findings from phase IV.

### Phase V: translation of the draft COACH IV tool

As the tool aims to be utilized in settings where the majority of respondents do not understand English, the COACH version IV tool was translated from English into Bangla (Bangladesh), Vietnamese (Vietnam), Lusoga (Uganda), isiXhosa (South Africa) and Spanish (Nicaragua) ahead of field-testing. The process of translation followed Brislin’s model which has been summarized by Yu et al. [48] and included *forward translation* by bilingual individual, *review* of the translated tool by monolingual reviewer, *backward translation* by bilingual individual and *comparison* of the original version and the backward translated version focusing on conceptual clarity. Hence, the English COACH version IV tool was carefully compared with each of the backward-translated versions in several rounds to identify where and why the versions did not conform. In all settings, the country-specific researcher(s) undertook the forward translation, whereas professional translators undertook the backward translation.

### Phase VI: investigating internal structure and reliability

To investigate internal structure, it is advised to have a sample of 100–200 eligible respondents [49]. In the assessment of internal structure and reliability, we strived to include an equal number of respondents across the three healthcare professional groups (CHWs, nurse/midwives and physicians) and the five countries. Hence, the translated version of COACH version IV was administered to eligible respondents in Bangladesh (*n* = 71), Vietnam (*n* = 195), Uganda (*n* = 134), South Africa (*n* = 162) and Nicaragua (*n* = 150) (see Table 2). Information about the project was given and informed consent obtained prior to participation. In addition to the 68 tested COACH version IV items, we included seven demographic questions. Participants filled in the questionnaire on paper. The exception to this was the CHW group in Nicaragua who requested that a data collector interview them and then fill in the form on their behalf.

**Table 2:** Demographic characteristics of study population phase VI

Country	Included respondents (*n*)	Excluded respondents (*n*)	Total (*n*)
Bangladesh	71	0	71
Vietnam	183	12	195
Uganda	134	0	134
South Africa	161	1	162
Nicaragua	141	9	150
Study population	690	22	712
Sex			
Female	508	16	524
Male	167	4	171
Missing	15	2	17
Age by group			
<25	47	1	48
25–29	93	0	93
30–34	121	2	123
35–39	81	2	83
40–44	103	1	104
45–49	85	4	89
50–54	86	3	89
55–59	48	5	53
≥ 60	17	3	20
Missing	9	1	10
Health professional category			
Physician	215	4	219
Nurses/midwives	247	0	247
CHWs	224	15	239
Missing	4	3	7

Data were entered manually in Excel or by using an online data capturing form. Cases that had ≥20 missing values on the 68 context items were deleted [50]. A principal component analysis (PCA), using listwise deletion, was used to extract the major contributing factors and a Varimax rotation (orthogonal) was performed using SPSS (version 20) to identify the common factors. Factors with eigenvalues greater than 1 were also extracted. A factor loading greater than 0.40 was regarded as ‘practically significant’ in accordance with Hair et al. [51]. The rationale for choosing PCA was that the assessment was of exploratory nature. To render partial drop-out, k-nearest neighbour imputation was undertaken [52]. Following exclusion (see Table 2), the total number of respondents was 690: Bangladesh (*n* = 71), Vietnam (*n* = 183), Uganda (*n* = 134), South Africa (*n* = 161) and Nicaragua (*n* = 141). The PCA was undertaken on the pooled dataset, per country and per health worker category. Factors were identified using the 1.0 eigenvalue cut-off rule and Scree test [33]. We agreed that the following criteria for acceptable level of internal structure and reliability should be met:

1. The item should have a factor loading >0.4 on the pooled dataset, and items belonging to the same theoretical dimension should load on the same factor.
2. The item should have a factor loading of >0.4, and items belonging to the same theoretical dimension should load on the same factor in the majority of settings (i.e. at least in three out of five settings).
3. The item should have a factor loading of >0.4, and items belonging to the same theoretical dimension should load on the same factor in the majority of health professional groups (i.e. at least in two of three health professional groups).
4. The factor should have a Cronbach’s Alpha coefficient of ≥0.7 [53].

The criteria assisted in the retention of items and thus established an acceptable internal structure of the tool. Following retention of items, we examined corrected total item correlation and average inter-item correlation [53, 54].

## Results

Findings from the six phases of development are presented below. Findings from each phase resulted in revisions on the tested COACH tool draft version that was then assessed in the next phase (see Fig. 1).

### Phase I: defining dimensions and developing a draft version of the COACH tool

The definition of the dimensions and development of corresponding items was an iterative process whereby we initially included all eight dimensions in the ACT (*n* = 58 items) as well as additional dimensions thought to be relevant for LMIC settings. Our initial discussions resulted in definitions of some of the original ACT dimensions remaining ‘intact’ whilst others were adapted and included (see Additional file 1). In some cases, for existing ACT dimensions, new items were developed that were relevant for LMIC settings. Finally, three new dimensions, *Organizational resources*, *Community engagement* and *Commitment*, believed to be of importance for LMICs were developed specifically for the COACH tool, and new items were also developed for these dimensions. For example, to capture the proposed new dimension *Commitment*, we reviewed literature and opted to use parts of the Organizational Commitment Questionnaire (OCQ) and the Affective Commitment Scale (ACS) [55, 56] to cover the given definition of the dimension (see Additional file 1). In this paper, items that were originally developed by us are referred to as the COACH items, whilst the items from established tools such as the ACT, OCQ and ACS are referred to using their original instrument abbreviation.

Each item on all dimensions measured the extent to which a respondent agreed or disagreed with the statement on a 5-point Likert-type scale (Strongly disagree, Disagree, Neither agree nor disagree, Agree or Strongly agree). The one exception to this was the *Sources of knowledge* dimension, which measured how often respondents used different sources of knowledge in a typical month which was rated using another Likert-type scale (Not available; Never, 0 times; Rarely, 1–5 times; Occasionally, 6–10 times; Frequently, 11–15 times; and Almost always, 16 times or more). In terms of general terminology used in the COACH tool, the ACT term ‘organization/unit’ was replaced by the term ‘unit’ as it was identified as the level of organizational context of interest.

### Phase II: testing content validity in country panels

For the content validity exercise with the English version of the COACH version I tool, none of the dimensions reached the generally accepted threshold scale-content validity index/average (S-CVI/Ave) ≥0.9 or scale-content validity index/universal agreement (S-CVI/UA) ≥0.8 in all of the four settings [45]. However, several dimensions reached S-CVI/Ave of 0.9 in one or more of the settings. Panel participants from all settings were in agreement regarding relevance of 31/94 items across all dimensions (I-CVI >0.78) (see Additional file 2).

In spite of the mixed results in the CVI assessment, panellists in all settings considered that all dimensions were relevant in the qualitative component of this phase. In particular, *Leadership*, *Resources* and *Work culture* were identified as important contextual influences that affect the implementation of EBPs in health settings. In addition to the dimensions presented to panellists in COACH version I tool, informal payment and nepotism were thought to be issues that influence the process of implementing EBPs in these settings. Examples of informal payment included the sale of drugs and services to patients that should be available free of charge. Participants also brought up the existence of ‘informal systems’ whereby health workers, primarily physicians, made decisions concerning healthcare delivery on the basis of payments into their own pocket, e.g. allowing one’s private patient to bypass the queue in a government healthcare facility.

The development of the COACH version II tool was based on findings from the content validity assessment from country panels. Some examples of revisions included the following. (1) Items relating to technological resources under *Sources of knowledge* were retained although they had not been found to be relevant as these resources are quickly becoming common in many parts of the world. (2) Under *Organizational resources*, items relating to *Financing* were added to assess an organization’s ability to autonomously manage their funds, as this had been identified as likely to impact upon the implementation of EBPs. (3) Items belonging to the dimension of *Feedback* were well understood and perceived as important but it was felt that the dimension had a broader focus and was more directed towards the continuous monitoring of services in order to inform implementation activities—thus, the dimension was renamed to *Monitoring services for action.* (4) As *Culture* and *Community engagement* were perceived as two important aspects of context, we developed additional items for these two dimensions. (5) *Formal* and *Informal interactions* were merged into one dimension of context, namely *Interaction between members of the unit.*

There was consensus that the language in the tool needed to be simplified for ease of understanding for all types of healthcare providers. Hence, during the development of COACH version II, we consulted the Language Centre at Stellenbosch University who converted the English version of the tool into plain language, which was deemed to be appropriate for the target audience’s average language proficiency and grasp of terminology.

### Phase III: testing content validity in an international panel

For the second set of content validity assessments with COACH version II, none of the concepts reached S-CVI/Ave ≥0.9 or S-CVI/UA ≥0.8. In total, 44 of the tested 78 items reached I-CVI of 0.78 (see Additional file 3). In order to keep the dimension of *Sources of knowledge*, where only one item reached the desired I-CVI of 0.78, we decided to keep all items with an I-CVI above 0.67 although this decision implied a diversion from the generally acceptable I-CVI score. The rationale for this decision was that several of the experts stated that some of the electronic sources of knowledge were currently not available in all the LMICs they had experiences from and thus not perceived as relevant. They did, however, also comment that these resources are very quickly becoming more common. Furthermore, we concluded that a change in I-CVI threshold should be consistent throughout the tool. Hence, in total, 63/78 items reached I-CVI of 0.67 (see Additional file 3). Based on comments made by the participating experts, some linguistic revisions were undertaken to single items [57]. Further development based on findings from phase III resulted in the COACH version III tool.

### Phase IV: investigating and analysing response process data

The response process investigation in South Africa revealed that most items were understandable without difficulty. Most items did not need any revisions. Minor revisions included changing an item such as *My unit has adequate space to provide services* to *My unit has enough space to provide healthcare services.* It was however noted that about 10 of the items were challenging for respondents to understand. For example, respondent reacted to items using words that were considered ambiguous. One such example was an item under the *Informal payment* dimension: *It is possible for staff to earn extra income from other work or engagements during ordinary working hours*. The concept of ‘other work engagements’ was perceived as too complicated, and respondents needed further explanation in order to respond to the item. In order to simplify the final version of that item, it became *Health workers are sometimes absent from work earning money at other places* [57].

Based on findings from the response process, further linguistic adaptations were made in collaboration with the Language Centre at Stellenbosch University. These adjustments resulted in the COACH version IV tool that was then considered ready for field-testing.

### Phase V: translation of the draft COACH IV tool

The COACH version IV tool in English and the five backward translated tools were carefully compared focusing on conceptual clarity in order to identify where and why the tools did not conform. One dimension that was challenging to translate was the one of *Informal payment*. As an example, the phenomenon in the introduction to the dimension was translated to *money and gift* (*‘envelope’ payments*) in Vietnam. Reaching to contextually adapted translations that reflected the intended construct needed thorough discussions.

### Phase VI: investigating internal structure and reliability

Following the administration of the translated version of COACH version IV in the five country settings, we investigated the internal structure and reliability of the tool. In conclusion, the analysis and parallel refining work resulted in the COACH version V having a good fit between the theoretical constructs and results from factor analysis. Cleaned data were merged from all settings into one file where descriptive statistics were examined and found satisfactory. The factor analysis revealed that an 11-factor structure accounted for 63.6 % of the variance in the pooled dataset. These 11 factors did relatively well represent the theoretical dimensions that the development of the instrument departed from (Table 3). None of the items cross-loaded >0.4 on two factors in the pooled dataset, but a few items cross-loaded >0.4 on two factors in the analysis made per country and healthcare professional group. Table 3 provides the result from the factor analysis and Cronbach’s alpha coefficient per theoretical dimension in the pooled data set. Table 4 provides a detailed description of the proportion of items reaching the set criteria for factor loadings per country and per professional group and summarizes the extent to which items in theoretical dimension loaded on the same factor in the different sub-analyses.

**Table 3:** Internal structure and internal consistency for COACH version V tool, phase VI

			Rotated component matrix^a^											Cronbach’s alpha	Corrected total item correlation	Average inter-item correlation
			1	2	3	4	5	6	7	8	9	10	11			
Resources	My unit has enough workers with the right training and skills to do everything that needs to be done	Human resources											0.84	0.84	0.40–0.66	0.32
	My unit has enough workers with the right training and skills to do their job in the best possible way	Human resources											0.86			
	My unit has enough space to provide healthcare services	Space											0.47			
	My unit has access to the transport and fuel that are needed to provide healthcare services	Communication and transport						0.66								
	My unit has access to the communication tools (e.g. telephones or radios) that are needed to provide healthcare services	Communication and transport						0.72								
	My unit receives money according to a budget	Financing						0.52								
	My unit has money that we can decide how to use	Financing						0.57								
	My unit has enough medicine to provide healthcare services	Medicines and equipment							0.80							
	My unit has enough functional equipment to provide healthcare services	Medicines and equipment							0.76							
	My unit has enough disposable medical equipment, such as syringes, gloves and needles to provide healthcare services	Medicines and equipment							0.76							
	If the workload increases, my unit can get additional resources such as medicine and equipment	Medicines and equipment							0.70							
Community engagement	In my unit, we ask community members what they think about the healthcare services that we provide					0.72								0.83	0.58–0.66	0.49
	In my unit, we listen to what community members think about the healthcare services we provide					0.72										
	In my unit, we have meetings with community members to discuss health matters					0.75										
	In my unit, we encourage community members to contribute to improving the health of the community					0.74										
	In my unit, we encourage other organizations to contribute to improving the health of the community					0.67										
Monitoring services for action	I receive regular updates about my unit’s performance based on information/data collected from our unit						0.70							0.84	0.57–0.70	0.53
	My unit discusses information/data from our unit in a regular, formal way, such as in regularly scheduled meetings						0.70									
	My unit regularly uses unit information/data to make plans for improving its healthcare services						0.67									
	My unit regularly monitors its work by comparing it with the unit’s action plans						0.70									
	My unit regularly compares its work with national or other guidelines						0.68									
Sources of knowledge	Clinical practice guidelines	Structural sources										0.78		0.69	0.38–0.49	0.31
	Other printed material for work (e.g. textbooks, journals)	Structural sources										0.73				
	In-service training/ workshops/courses	Structural sources										0.69				
	The Internet	E-health						0.71								
	Electronic decision support (e.g. mobile phone applications or other electronic devices to assist with care and decision-making)	E-health						0.65								
Commitment to work	I am proud to work in this unit.									0.70				0.76	0.55–0.62	0.52
	I am satisfied to work in this unit.									0.76						
	I feel encouraged to do my very best at work.									0.72						
Work culture	My unit is willing to use new healthcare practices such as guidelines and recommendations	Culture of learning and change			0.69									0.83	0.56–0.65	0.45
	My unit helps me to improve and develop my skills	Culture of learning and change			0.57											
	I am encouraged to seek new information on healthcare practices	Culture of learning and change			0.75											
	My unit works for the good of the clients and puts their needs first	Culture of responsibility			0.65											
	Members of the unit feel personally responsible for improving healthcare services	Culture of responsibility			0.59											
	Members of the unit approach clients with respect	Culture of responsibility			0.54											
Leadership	I trust the unit leader.		0.59											0.89	0.61–0.80	0.59
	The leader handles stressful situations calmly.		0.80													
	The leader actively listens, acknowledges, and then responds to requests and concerns.		0.82													
	The leader effectively resolves any conflicts that arise.		0.80													
	The leader encourages the introduction of new ideas and practices.		0.75													
	The leader makes things happen.		0.73													
Informal payment	Clients must always give informal payment to health workers to access healthcare services	Informal payment		0.78										0.77	0.31–0.60	0.32
	Clients are treated more quickly if they make informal payments to health workers	Informal payment		0.83												
	Medicines or equipment that should be available for free to clients have been sold in my unit	Informal payment		0.78												
	Health workers are sometimes absent from work earning money at other places	Informal payment		0.73												
	Health workers in my unit give healthcare services to friends and family first	Nepotism		0.68												
	Health workers in my unit give jobs or other benefits to friends and family first	Nepotism		0.64												
	Efforts are made to stop clients from providing informal payment to get appropriate healthcare services	Accountability									0.87					
	Efforts are made to stop health workers from asking clients for informal payment	Accountability									0.86					

Extraction method: principal component analysis. Rotation method: Varimax with Kaiser Normalization

^a^Rotation converged in eight iterations

**Table 4:**
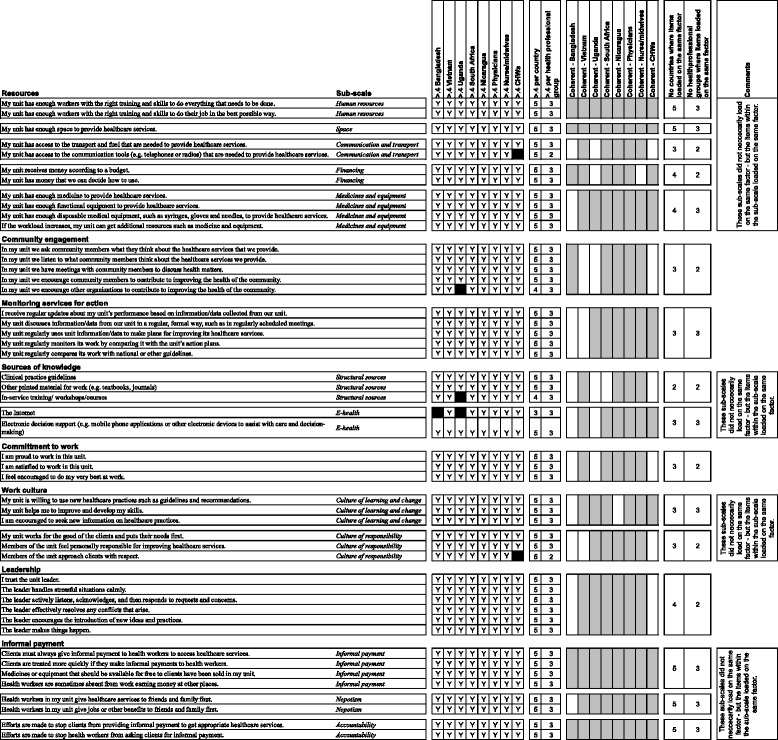
Summary internal structure analysis per country and health professional group, phase VI

Y = item loading >0.4

∎ = item loading <0.4

Reliability of the COACH version V tool was examined, and all dimensions but one reached acceptable Cronbach’s alpha levels of ≥0.70 (ranging between 0.76 and 0.89) [53], whilst *Sources of knowledge* reached a Cronbach’s alpha of 0.69. Corrected total item correlation and average inter-item correlation >0.3 was judged as good [53, 54]. All items in the dimensions had a corrected total item correlation of >0.3, and all dimensions had an average inter-item correlation of >0.3 (Table 3).

To summarize the field test, the COACH tool was investigated for internal structure in (1) the five different settings, (2) in three professional groups (pooled from the different settings) and (3) on the pooled dataset. The investigation of validity and reliability reduced the number of items from 67 to 49 measuring eight hypothesized contextual dimensions: *Resources*, *Community engagement*, *Monitoring services for action*, *Sources of knowledge*, *Commitment to work*, *Work culture*, *Leadership and Informal payment.* In applying our criteria for acceptable internal structure, the dimension of *Interaction among people* was excluded. Cronbach’s alpha, corrected total item correlation and average inter-item correlation provided evidence of reliability for the proposed eight-factor structure. As a consequence of the development process, the definitions of the dimensions were carefully scrutinized in the end of the project. The final definitions of dimensions are found in Table 5, which constitutes the final COACH (version V) tool (see Tables 3 and 4). The complete tool is available (see http://www.kbh.uu.se/IMCH/COACH).

**Table 5:** Definitions of dimensions of COACH tool version V

Dimension	Definition
Organizational resources	The availability of resources that allow an organization (unit) to adapt successfully to internal and external pressures
Community engagement	The mutual communication, deliberation and activities that occur between community members and an organization (unit)
Monitoring services for action	The process of using locally derived data to assess performance and plan how to improve outcomes in an organization (unit)
Sources of knowledge	The availability and use of sources of knowledge in an organization (unit) to facilitate best practice
Commitment to work	The individual’s identification with and involvement in a particular organization (unit)
Work culture	The way ‘we do things’ in an organization (unit) reflecting a supportive work culture
Leadership	The actions of a formal leader in an organization (unit) to influence change and excellence in practice achieved through clarity and engagement
Informal payment	Payments or benefits given to individual(s) in an organization (unit), which are made outside the officially accepted arrangements, to acquire an advantage or service

## Discussion

We have developed a new tool for assessing the context of healthcare organizations in LMIC settings with promising psychometric characteristics that provides insight into factors influencing the implementation of EBPs. The development of the COACH tool resulted in a tool covering eight dimensions of context and comprising 49 items. Currently, the tool is available in six languages. Whilst many of the dimensions of context that are central in high-income settings are also relevant in LMIC, also other dimensions (such as *Informal payment*, *Commitment to work* and *Community engagement*) were found to have particular resonance in LMICs and which are the focus of this discussion.

*Informal payment* is an influential factor in the implementation and provision of EBPs. The shifting of priorities in healthcare delivery, such as prioritizing the provision of healthcare to clients who can offer payment as opposed to those in greatest need has been reported [58–61]. It is important to get a better understanding of how much of a barrier informal payment is, especially in settings where policies of free provision of health services for vulnerable groups exist [62]. Informal payment may lead to limited availability of health services for those in need but may also be an important aspect of health workers’ motivation and retention in settings with widespread demoralization and demotivation due to low wages and poor human resource management [62, 63].

Previously, we have described that some health workers in Uganda had to provide informal payments in order to obtain employment, and then again to get on the payroll once having acquired a position [29]. In that case, informal payment is imposed from above and there is a risk that such behaviour will continue down the health system’s hierarchy [64]. In the settings where the COACH tool was developed, the Global Corruption Barometer 2013 has found that 9–43 % of respondents had paid bribes to medical and health services within the last 12 months, and 33–58 % perceived that medical and health services are corrupt (data for Nicaragua was not available) [65]. The effects of informal payments and nepotism remain silent and provide an ongoing threat to achieving continued progress in global health [66].

A further aspect of context that is included in the COACH tool but that has not been a part of other tools is *Commitment to work*. A recent review focusing on mechanisms for the successful implementation of support strategies for healthcare practitioners in rural and remote contexts found that strong organizational commitment is linked to greater participation levels, change in organizational culture, sustainable programmes and improved patient outcomes and quality of provided services [35]. From an equity perspective, poor clients in high-mortality countries often experience neglect, abuse, and marginalization by the health system by overworked and demotivated and uncommitted health workers [63, 67]. Motivation and staff satisfaction have been shown to be critical elements in improving the efficiency and effectiveness of health system performance, amongst mid-level providers in Malawi [68]. Commonly, competencies are assessed ahead of implementing EBPs. Mapping of individual health workers organizational commitment, motivation and barriers to good performance would also benefit our understanding of performance.

Another characteristic of context that was important in study countries was *Community engagement*. Central to the WHO health system building blocks model is the role of people not only as beneficiaries, but also as active drivers of the health system [43]. For example, strengthening the linkage between primary health centres and the community and reinforcing the power and involvement of community members in health service delivery appear to have contributed to a 33 % reduction in under-five mortality in Uganda [69], a 50 % reduction in neonatal mortality in Vietnam [70] and a significant reduction in child mortality in Nicaragua [71].

The impact of *Work culture* on the implementation of EBPs has led to calls to understand the culture in which a particular innovation will be implemented prior to its implementation [72–74]. In the case of the COACH tool, two separate aspects of culture were found to influence the implementation of EBPs: (1) working within a culture valuing learning and (2) harbouring a sense of responsibility to improve healthcare. Similar to other studies [74–77], we found that workplace culture in general, and teamwork in particular, was perceived to be an important contributing factor for health worker motivation, with poor teamwork being associated with difficulties in implementing change.

*Leadership* and the ability of the leader to create an environment of teamwork was emphasized by panellists in phase II–III. In addition, trust was described as a factor influencing leaders’ ability to form a culture where the implementation of EBPs would occur more effectively. Individuals’ trust in supervisors was related to personal behaviour, and supervisors’ actions might affirm or undermine the trust in the organization [78]. Panellists believed that lack of trust, either personal or professional, affected the organization’s readiness to adapt to changes. The importance of leadership and teamwork for the implementation of EBPs to occur has been described in other studies from low-income settings [77, 79–81].

## Methodological considerations

Challenges in the development of a generic instrument to assess context in LMIC included whether the instrument was broad enough to include common aspects of context, but also specific with regard to including contextual elements of importance for implementation. Throughout the project, the research team has strived not to be too radical and delete items too quickly, but rather keep items and reduce the number of items with caution. One reflection of this is the lowering of cut-off for I-CVI from 0.78 to 0.67 during phase III in order to keep several of the items under the *Sources of knowledge* dimension. Different from the other dimensions asking about agreement, this dimension asks for *how often different sources of knowledge are used in a normal month*. It should however be noted that the experts were not seeing the answering options the way that they are presented in the tool—instead, they were subjected to a scale where they could rate their level of agreement with the relevance of the item. As some experts noted that these sources of knowledge were not currently available, without knowing that *Resource not available* is one of the answering options in the tool, we judged that the experts might have rated the relevance of these items different had they known that the scale had that option.

With regard to the findings for the two phases of content validity assessment (phase II–III), it was not surprising that none of the dimensions reached S-CVI/UA ≥0.8 across all settings since it requires unanimity amongst all raters. Furthermore, S-CVI/UA has been criticized to be ‘too stringent’, especially when used with larger groups [44].

Careful translations and backward translation was of uttermost importance. In translating the English version of the tool, we strived to be loyal to the meaning of the text (semantics) and to enable adaption for the translation to fit (context) [48]. In order to do this, it is essential that the parties working with the translation have a common language and that the communication allows for semantic discussions throughout the process. Although the translation process was time consuming, its importance was confirmed by the factor analysis which showed that the items within each dimension fitted well together across all countries, suggesting that the translated items measure the same concepts.

The *Sources of knowledge* dimension was not rated as relevant by international experts and lead us to lower the level of acceptable I-CVI score. One reason for this was low scoring of e-health and m-health items due to the unavailability of this type of technical devices. We, however, opted to keep these items as the development and utilization of technology is rapidly changing. We aimed to include dimensions reaching a Cronbach’s alpha coefficients of ≥0.7. Although the dimension of *Sources of knowledge* did not meet this criterion, the availability and usage of information were considered important, and as there is no cut-off that exactly determines the reliability of instrument dimensions, we decided to remain this dimension with its five items for future evaluations of its reliability.

Although there were many similarities in the psychometric evaluation of the tool across settings and occupational groups, some differences were also present on country/professional level. Similarly, some items cross-loaded on analysis per country and health professional group. The items that were cross-loading were, however, not consistent between groups (countries and health professional groups), and we therefore decided to retain the cross-loading items based on results from the analysis made on the pooled dataset. Further evaluation of the tool will show if keeping these cross-loading items was an appropriate decision.

## Conclusion

The newly developed COACH tool specifically aims to assess the context of healthcare organizations in LMIC settings, thereby providing increased potential for insight into factors influencing the implementation of EBPs in these settings. Whilst we departed from the ACT in developing a tool that would be suitable in LMICs, the COACH tool shares some content with the ACT and is a derivative product of the ACT. Although many of the organizational context concepts recognized in high-income healthcare settings were found to be relevant also in LMICs, we identified additional aspects of context significant for the implementation of EBPs in LMICs. We foresee alternative ways of applying the COACH tool as means of characterizing context ahead of implementing health interventions, as a method for tailoring an implementation strategy to suit a certain context and for deepening the understanding of the outcomes of implementation efforts. All these applications have the potential to generate better understanding of the process of implementing EBPs.

## Additional files

Additional file 1: **Definitions of dimensions of COACH tool draft version I, phase I.** (PDF 446 bytes)
Additional file 2: **Outcomes of content validity assessment in Bangladesh, Vietnam, Uganda and Nicaragua, phase II.** (PDF 153 kb)
Additional file 3: **Outcomes of content validity assessment amongst international experts, phase III.** (PDF 6.55 kb)

## Abbreviations

ACS: Affective Commitment Scale
ACT: Alberta Context Tool
CHW: community health worker
COACH: Context Assessment for Community Health
EBP: evidence-based practice
iccdr,b: International Centre for Diarrhoeal Disease Research, Bangladesh
I-CVI: item-content validity index
OCQ: Organizational Commitment Questionnaire
PARIHS: Promoting Action on Research Implementation in Health Services
PCA: principal component analysis
S-CVI/Ave: scale-content validity index/average
S-CVI/UA: scale-content validity index/universal agreement
WHO: World Health Organization
