# Supplementary material for: Health system context and implementation of evidence-based practices—development and validation of the Context Assessment for Community Health (COACH) tool for low- and middle-income settings
Source: Implement Sci. 2015 Aug 15;10:120. doi: 10.1186/s13012-015-0305-2 (PMC4537553; doi:10.1186/s13012-015-0305-2)
Supplement: Additional file 1: — Definitions of dimensions of COACH tool draft version I, phase I. (PDF 446 bytes) [file 13012_2015_305_MOESM1_ESM.docx]

Additional file 1: Definitions of dimensions of COACH tool draft version I, phase I

| **Tool** | **Dimension** | **Definition** | **Definition procedure** |
| --- | --- | --- | --- |
| **ACT** | *Leadership* | The actions of formal leaders in an organization (unit) to influence change and excellence in practice, items generally reflect emotionally intelligent leadership. | The relevance of the ACT construct and definition of ‘Leadership’ was thoroughly discussed within the international research group. The construct was perceived as very important and the definition passable in a low- and middle-income setting. |
| **COACH** | *Leadership* | The actions of formal leaders in a unit to influence change and excellence in practice, items generally reflect emotionally intelligent leadership |  |
| **ACT** | *Culture* | The way that ‘we do things’ in our organizations and work units, items generally reflect a supportive work culture. | The relevance of the ACT construct and definition of ‘Culture’ was thoroughly discussed within the international research group. The construct was perceived as very important and the definition passable in a low- and middle-income setting. |
| **COACH** | *Culture* | The way that ‘we do things’ in our units, items generally reflect a supportive work culture. |  |
| **ACT** | *Evaluation* | The process of using data to assess group/team performance and to achieve outcomes in organizations or units. | The relevance of the ACT construct and definition of ‘Evaluation’ was thoroughly discussed within the international research group. Commonly, routine data gathering and quality of collected data is substandard in the settings that the COACH is developed for. The research group therefore had a discussion and decided that the term ‘data’ in the ACT definition was replaced by the broader term ‘information/data’.  Secondly, the experience of the research group was that emphasis of the evaluation component should be on the *usage* of locally gathered information to *drive change*. Discussions lead to the revision of the construct from ‘Evaluation’ to ‘Monitoring services for action’. |
| **COACH** | *Monitoring services for action* | The process to assess group/team performance and to achieve outcomes in units by using local health service delivery information. |  |
| **ACT** | *Formal interactions* | Formal exchanges that occur between individuals working within an organization (unit) through scheduled activities that can promote the transfer of knowledge. | The relevance of the ACT construct and definition of ‘Formal interaction’ was thoroughly discussed within the international research group. The concept was found to be very relevant and interesting to explore in the settings that COACH is intended for. In contrast to the ACT definition, focusing on promoting the ‘transfer of knowledge’ the research group decided to focus on the ‘transfer of knowledge and skills’. |
| **COACH** | *Formal interactions* | Formal exchanges that occur between individuals working within a unit through scheduled activities that can promote the transfer of knowledge and skills. |  |
| **ACT** | *Social Capital* | The stock of active connections among people. These connections are of three types: bonding, bridging, and linking. | The relevance of the ACT construct and definition of ‘Informal interaction’ and ‘Social capital’ was thoroughly discussed within the international research group and found to be relevant. However, discussions lead to the group merging these two constructs into one ‘Connection among people’, reflecting the level of the interpersonal relationship in relation to the informal transfer of evidence-based practices. |
| **ACT** | *Informal interactions* | Informal exchanges that occur between individuals working within an organization (unit) that can promote the transfer of knowledge. |  |
| **COACH** | *Connections among people* | The stock of active connections among people that can promote the informal transfer of evidence‐based practices. These connections are of three types: bonding, bridging and linking. |  |
| **ACT** | *Structural/*  *electronic resources* | The structural and electronic elements of an organization (unit) that facilitate the ability to assess and use knowledge. | The ACT definition of ‘Structural and electronic resources’ was circulated among the research group members. The construct was perceived as very important.  When we discussed, the next coming construct of ‘Organizational resources’ was perceived to stand for the ‘Structural’ part of resources needed to implement new knowledge. In addition, we found the term ‘Electronic resources’ too narrow as the majority of items in the construct were not electronic. To simplify, we therefore decided to rename this construct. |
| **COACH** | *Sources of knowledge* | The structural and electronic elements of a unit that facilitate the ability to assess and use knowledge. |  |
| **ACT** | *Organizational slack* | The cushion of actual or potential resources, which allows an organization (unit) to adapt successfully to internal pressures for adjustments or to external pressures for changes. | The ACT definition of ‘Organizational resources’ was circulated among the research group members. The construct was perceived as very important.  Following discussions in the group, the definition of the construct shifted from emphasizing ‘slack’ in terms of resources to *the real availability of needed recourses*. The construct should still reflect ‘slack’, although *less prominent*.  From qualitative research and lived experience, the research group found that there was a need to investigate health workers perception of the availability of resources in general. Therefore, the sub-groups of items were broadened from the ACT version of ‘staffing, space and time’ to include ‘staff, space, time, communication and transport, drugs, equipment and supplies’. |
| **COACH** | *Organizational resources* | The availability of resources (staff, space, time, communication and transport, drugs, equipment and supplies) that allows a unit to adapt successfully to internal pressures for adjustments or to external pressures for change. |  |
| **COACH** | *Community engagement* | The mutual communication, deliberation and activities that occur between community members and units. | The experiences from the research group members was that the involvement of the community on the provided health services as well as a growing ‘demand for service’ in these settings will influence the knowledge translation process. This dimension is not found in the ACT. |
| **COACH** | *Commitment* | The intellectual and emotional binding to the mission of the unit. | The experiences from the research group members was that a low uptake of new practices is influenced by the motivation and commitment among health workers in the unit. This dimension is not found in the ACT. The definition of the dimension was instead influenced by the ACS and the OCQ. |
