# Supplementary material for: Health system context and implementation of evidence-based practices—development and validation of the Context Assessment for Community Health (COACH) tool for low- and middle-income settings
Source: Implement Sci. 2015 Aug 15;10:120. doi: 10.1186/s13012-015-0305-2 (PMC4537553; doi:10.1186/s13012-015-0305-2)
Supplement: Additional file 2: — Outcomes of content validity assessment in Bangladesh, Vietnam, Uganda and Nicaragua, phase II. (PDF 153 kb) [file 13012_2015_305_MOESM2_ESM.docx]

Additional file 2: Outcomes of content validity assessment in Bangladesh, Vietnam, Uganda and Nicaragua, phase II

| **Dimension** | **Total number of items (n=94)** | **No of relevant items, I-CVI ≥ 0.78, in all four settings (n=31)** | **No of relevant items, I-CVI ≥ 0.78, in three out of four settings (n=67)** |
| --- | --- | --- | --- |
| Leadership | ACT: 6 items  COACH: 3 items | ACT: 2 items  COACH: 3 items | ACT: 5 items  COACH: 3 items |
| Culture | ACT: 6 items  COACH: 6 items | ACT: 3 items  COACH: 2 items | ACT: 5 items  COACH: 4 items |
| Feedback | ACT: 6 items  COACH: 2 items |  | ACT: 4 items  COACH: 5 items |
| Connections among people | ACT: 6 items  COACH: 1 item | ACT: 4 items | ACT: 6 items  COACH: 1 item |
| Formal interactions | ACT: 4 items  COACH: 2 items | ACT: 1 item  COACH: 2 items | ACT: 2 items  COACH: 2 items |
| Informal interactions | ACT: 10 items | ACT: 2 items | ACT: 4 items |
| Structural/electronic resources | ACT: 10 items*  COACH: 1 item | ACT: 1 item | ACT: 3 items  COACH: 1 item |
| Organizational resources | ACT: 10 items  COACH: 10 items | ACT: 5 items  COACH: 3 items | ACT: 6 items  COACH: 8 items |
| *Staffing* | ACT: 3 items  COACH: 2 items | ACT: 2 items | ACT: 2 items  COACH: 2 items |
| *Space* | ACT: 3 items  COACH: 1 item | ACT: 2 items  COACH: 1 item | ACT: 2 items  COACH: 1 item |
| *Time* | ACT: 5 items  COACH: 1 item | ACT: 1 item | ACT: 2 items  COACH: 1 item |
| *Communication and transport* | COACH: 3 items |  | COACH: 2 items |
| *Drugs, equipment and supplies* | COACH: 3 items | COACH: 2 items | COACH: 2 items |
| Community engagement | COACH: 3 items | COACH: 1 items | COACH: 3 items |
| Commitment | OQC: 4 items  ACS: 4 items | OQC: 2 items | OQC: 3 items  ACC: 2 items |
| **TOTAL** | **ACT: 58 items**  **COACH: 28 items**  **OQC: 4 items**  **ACS: 4 items** | **ACT: 18 items**  **COACH: 11 items**  **OCQ: 2 items** | **ACT: 35 items**  **COACH: 27 items**  **OQC: 3 items**  **ACS: 2 items** |

*One ACT item missing

In this article, items that were originally developed by the Context Assessment for Community Health (COACH) group are referred to as COACH items, while the items originating from established tools such as the Alberta Context Tool (ACT) [[1](#_ENREF_1)], the Organizational Commitment Questionnaire (OCQ) [[2](#_ENREF_2)] and the Affective Commitment Scale (ACS) [[3](#_ENREF_3)] are referred to using their original instrument abbreviation.

**References**

1. Estabrooks, C.A., et al., *Development and assessment of the Alberta Context Tool.* BMC Health Serv Res, 2009. **9**: p. 234.

2. Mowday, R.T., R.M. Steers, and L.W. Porter, *Measurement of Organizational Commitment.* Journal of Vocational Behavior, 1979. **14**(2): p. 224-247.

3. Allen, N.J. and J.P. Meyer, *The measurement and antecedents of affective, continuance and normative commitment to the organization.* The British Psychological Society, 1990. **63**: p. 1-18.
