# Supplementary material for: Health system context and implementation of evidence-based practices—development and validation of the Context Assessment for Community Health (COACH) tool for low- and middle-income settings
Source: Implement Sci. 2015 Aug 15;10:120. doi: 10.1186/s13012-015-0305-2 (PMC4537553; doi:10.1186/s13012-015-0305-2)
Supplement: Additional file 3: — Outcomes of content validity assessment amongst international experts, phase III. (PDF 6.55 kb) [file 13012_2015_305_MOESM3_ESM.docx]

Additional file 3: Outcomes of content validity assessment amongst international experts, phase III

| **Dimension** | **Total number of items (n=78)** | **No of relevant items, I-CVI ≥ 0.78, (n=44)** | **No of relevant items, I-CVI ≥ 0.67, (n=63)** |
| --- | --- | --- | --- |
| Organizational resources | ACT: 5 items  COACH: 14 items | ACT: 5 items  COACH: 7 items | ACT: 5 items  COACH: 9 items |
| *Human resources* | ACT: 3 items  COACH: 3 items | ACT: 3 items | ACT: 3 items |
| *Space* | ACT: 2 items  COACH: 1 item | ACT: 2 items | ACT: 2 items |
| *Communication and transport* | COACH: 3 items | COACH: 2 items | COACH: 3 items |
| *Medicines, equipment and consumables* | COACH: 4 items | COACH: 4 items | COACH: 4 items |
| *Financing* | COACH: 3 items | COACH: 1 items | COACH: 2 items |
| Community engagement | COACH: 5 items | COACH: 4 items | COACH: 5 items |
| Monitoring services for action | ACT: 5 items  COACH: 3 items | ACT: 1 item  COACH: 1 item | ACT: 4 items  COACH: 1 items |
| Sources of knowledge | ACT: 7 items | ACT: 1 item | ACT: 4 items |
| Interaction between members of the unit | ACT: 4 items  COACH: 1 item | ACT: 2 items | ACT: 3 items |
| Commitment | OQC: 3 items  ACS: 3 items | OQC: 3 items  ACS: 2 items | OQC: 3 items  ACS: 2 items |
| Culture | ACT: 4 items  COACH: 9 items | ACT: 4 items  COACH: 8 items | ACT: 4 items  COACH: 8 items |
| Leadership | ACT: 3 items  COACH: 2 items | ACT: 2 items  COACH: 1 items | ACT: 3 items  COACH: 2 items |
| Informal payment | COACH: 10 items | COACH: 3 items | COACH: 10 items |
| **TOTAL** | **ACT: 28 items**  **COACH: 44 items**  **OQC: 3 items**  **ACS: 3 items** | **ACT: 15 items**  **COACH: 24 items**  **OQC: 3 items**  **ACS: 2 item** | **ACT: 23 items**  **COACH: 35 items**  **OQC: 3 items**  **ACS: 2 item** |

In this article, items that were originally developed by the Context Assessment for Community Health (COACH) group are referred to as COACH items, while the items originating from established tools such as the Alberta Context Tool (ACT) [[1](#_ENREF_1)], the Organizational Commitment Questionnaire (OCQ) [[2](#_ENREF_2)] and the Affective Commitment Scale (ACS) [[3](#_ENREF_3)] are referred to using their original instrument abbreviation.

**References**

1. Estabrooks, C.A., et al., *Development and assessment of the Alberta Context Tool.* BMC Health Serv Res, 2009. **9**: p. 234.

2. Mowday, R.T., R.M. Steers, and L.W. Porter, *Measurement of Organizational Commitment.* Journal of Vocational Behavior, 1979. **14**(2): p. 224-247.

3. Allen, N.J. and J.P. Meyer, *The measurement and antecedents of affective, continuance and normative commitment to the organization.* The British Psychological Society, 1990. **63**: p. 1-18.
